# Indicators of sustainable capacity building for health research: analysis of four African case studies

**DOI:** 10.1186/1478-4505-9-14

**Published:** 2011-03-28

**Authors:** Imelda Bates, Miriam Taegtmeyer, S Bertel Squire, Daniel Ansong, Bertha Nhlema-Simwaka, Amuda Baba, Sally Theobald

**Affiliations:** 1 Liverpool School of Tropical Medicine, Liverpool, UK; 2 School of Medical Sciences, Kwame Nkrumah University of Science and Technology, Kumasi, Ghana; 3 Research for Equity and Community Health Trust. Lilongwe, Malawi; 4 Institut Panafricain de Santé Communautaire, Democratic Republic of Congo

## Abstract

**Background:**

Despite substantial investment in health capacity building in developing countries, evaluations of capacity building effectiveness are scarce. By analysing projects in Africa that had successfully built sustainable capacity, we aimed to identify evidence that could indicate that capacity building was likely to be sustainable.

**Methods:**

Four projects were selected as case studies using pre-determined criteria, including the achievement of sustainable capacity. By mapping the capacity building activities in each case study onto a framework previously used for evaluating health research capacity in Ghana, we were able to identify activities that were common to all projects. We used these activities to derive indicators which could be used in other projects to monitor progress towards building sustainable research capacity.

**Results:**

Indicators of sustainable capacity building increased in complexity as projects matured and included

- early engagement of stakeholders; explicit plans for scale up; strategies for influencing policies; quality assessments (*awareness and experiential stages)*

- improved resources; institutionalisation of activities; innovation *(expansion stage)*

- funding for core activities secured; management and decision-making led by southern partners *(consolidation stage)*.

Projects became sustainable after a median of 66 months. The main challenges to achieving sustainability were high turnover of staff and stakeholders, and difficulties in embedding new activities into existing systems, securing funding and influencing policy development.

**Conclusions:**

Our indicators of sustainable capacity building need to be tested prospectively in a variety of projects to assess their usefulness. For each project the evidence required to show that indicators have been achieved should evolve with the project and they should be determined prospectively in collaboration with stakeholders.

## Background

Capacity building is strengthening the 'ability of individuals, organisations or systems to perform appropriate functions effectively, efficiently and sustainably' [1] and it is an integral component of many health development projects. Despite an increasing literature about the theory of evaluating capacity building [2,3] there is very little published evidence about how to monitor its effectiveness in practice. The goal of capacity building is to enable organisations to be adaptable and solve problems to achieve sustainability. The lack of published examples of practical evaluation indicators hinder our ability to determine whether resources invested in capacity building are being used effectively to achieve sustainability.

Indicators used for monitoring and evaluation are often based on the requirements of donors or auditors, and are not used for learning, strategic planning, enhancing performance and decision-making [4,5]. It has been difficult to devise generic indicators for monitoring and evaluating capacity building [4] because each project is unique. Strengthening the capacity of health systems is closely linked to building research capacity because high quality research is essential to identify and prioritise health needs, and to develop appropriate strategies to improve health outcomes. In this study we were particularly interested in examining the development of capacity for research and implementation in projects which originally had a research focus.

Indicators that have been used to measure changes in research capacity range from low level 'process' measurements (e.g. number of MSc or PhD scholarships, or grants awarded) to slightly higher 'impact' measurements (e.g. PhDs completed, number of publications or programmes led by developing country partners). Capacity (for example strengthened systems or creation of public value) is distinct from capabilities [5] and indicators generally do not reflect complex capabilities such as the degree of autonomous leadership by southern institutions or the potential for sustainability.

The aim of our study was to develop indicators that could be used prospectively by project managers and funders to monitor progress towards achieving sustainable health capacity. We used some of the authors' own projects from Africa as case studies. These projects were selected because they had all achieved sustainable capacity for research and implementation. We chose to use case studies because they are an effective way of understanding and identifying generic lessons (i.e. aspects that may be transferable) from complex and unique contexts [6-8]. We derived monitoring indicators from activities which were common to all the case studies so that they would be applicable to other contexts. We used the differences between the case studies to highlight how these indicators might be influenced by different contexts.

## Methods

### Selection of case studies

Members of the Liverpool School of Tropical Medicine's Global Health Development Group, who have extensive multi-disciplinary experience of working in partnership in many countries in sub-Saharan Africa, provided eleven case studies for consideration for the study. To minimise bias, the group devised selection criteria for selecting case studies. All case studies had to meet all of the following criteria. They should have:

• developed out of research projects that did not have capacity building as their primary objective (to reflect the usual 'real-life' situation)

• incorporated the three key strategies for effective capacity building (see below) [9]

• resulted in programmes that were led and managed by Southern partners, and had funding for core costs (e.g. staff, facilities, utilities) which was independent of the original project donor (i.e. they were sustainable)

The three key strategies for sustainable capacity building are:

• *A phased approach *- engage stakeholders from the start; begin with small, carefully monitored pilot projects designed to fill identified capacity gaps; expand gradually within a well-defined strategy and action plan [1]

• *Strengthen existing processes *- harmonise the new programme with existing systems and resources; avoid creating parallel systems [10]

• *Partnerships for problem solving - *ensure local ownership; partners should have a common purpose, shared responsibilities and obligations, and clearly delineated roles; mechanisms for sustainability are built in from the outset [11,12].

In common with most research projects, these case studies did not have capacity development as a primary objective. However, the researchers were aware that it would be necessary to build capacity in order to implement the results of the research. Since the selected case studies had the potential to be scaled up, capacity development was a secondary objective in all the case studies.

To ensure our indicators would be applicable to a wide range of contexts each case study was located in a different African country, and each focused on a different health topic and operated at a different tier of the health service. The four case studies (CS) that best matched the criteria were:

CS1: Improving evidence-based health care in Ghana

CS2: HIV voluntary counselling and testing services in Kenya

CS3: Effect of poverty on access to TB services in Malawi

CS4: Strategies to promote community health in the Democratic Republic of Congo (DRC)

### Analysis of case studies

To analyse the case studies we used a framework designed to evaluate a health research capacity building programme in Ghana [9] (table 1). The framework divided projects into four phases - awareness, experiential, expansion and consolidation. Although these phases are presented in a linear fashion, in practice they are often overlapping with no definitive marker of progression between the phases. Information concerning the goal of the original project and the capacity building activities that occurred in each of the four project phases was mapped onto the cells in the framework. For each phase there was a space in the framework to enter the 'indicators of progress' that had been used in the project. The information was extracted from each of the case studies by dialogue between the authors, a process which enhanced the trustworthiness of the analysis [13]. Through this mapping process we were able to identify activities in each phase that were the same in all projects, and those that were different, and to list the indicators used to assess progress at each phase.

**Table 1:** Framework for monitoring and evaluating capacity building programmes

Phase	Indicators of progress (outcomes/outputs and approximate date achieved/anticipated)			
	**Case study 1 KATH, Ghanaian teaching hospital**	**Case study 2 Kenyan NGO LVCT**	**Case study 3 Malawian Research Unit REACH Trust**	**Case Study 4 DRC Research and Training. IPASC**

Aim of original project	To promote generation of local evidence to improve health care	To scale up access to HIV counselling and testing in primary health care centres	To develop evidence on equity, poverty and access to TB services in Malawi.	To understand the health needs of the community and develop context specific responses

Aim of capacity building component	Improve ability of teaching hospital to sustainably deliver and manage research skills course to UK standards without external resources	Improve ability of health care facility teams to deliver quality assured HIV counselling and testing and contribute to research findings	To build research skills in equity analysis and multi-method research to develop policy-relevant research	To provide training in community health grounded in context for different cadres

**Capacity building activities**				

Awareness phase "planning, awareness raising"	LSTM and KATH/KNUST jointly commit funds to improve capacity for conducting and using research Framework for monitoring progress developed	High HIV care burden in health care facilities with little knowledge of HIV status Recognition of lack of evidence about feasibility of this approach	Recognised need for operational research to guide NTP priorities Collaboration between NTP, LSTM and University of Malawi Obtained project funding	Recognised need for research, training and infrastructure development appropriate for rural, conflict/post-conflict DRC.
*Timing (months from start)*	*Started 2002 (0-12)*	*Started 2001 (0-18)*	*Started 1999 (0-1)*	*Started 1992 (0-36)*

Experiential phase "start up, testing"	UK off-site Diploma (DPDM) established in Ghana for all KATH health professionals Institutional research support services increased (e.g. internet access, research office established, earmarked local project funds); creation of faculty team	33 primary health facilities provide counselling and testing Kenyan NGO (LVCT) established for technical assistance to government to achieve scale up Research findings inform Kenyan guidelines and training	Studies conducted and fed into NTP policy and practice through Technical Working Groups First round of staff get Masters by Research from University of Malawi	IPASC is launched First graduates get degrees IPASC staff trained at LSTM on masters and PhD programmes
*Timing (months from start)*	*(9-36)*	*(12-36)*	*(12-24)*	*(24-108)*

Expansion phase "scale up, innovation"	Sustainable funding from MoH KATH fund quality assurance by LSTM Faculty for DPDM established with dedicated administration team First paper published by DPDM graduate	NGO expands to incorporate other post rape care, services for the disabled and for vulnerable groups Range of donors broadened and core funds increased First papers published	New staff recruited and research portfolio broadens to include HIV. Range of donors broadens and includes MoH funding Malawian director appointed; technical assistance from LSTM Malawian first author papers published	New courses established Range of donors broadened Became part of the EQUINET network Obtained funding to expand research DRC first author paper published
*Timing (months from start)*	*(24-60)*	*(30-72)*	*(40-60)*	*(108-192)*

Consolidation phase "sustainability, autonomy"	DPDM run entirely by KATH tutors; LSTM monitor quality Research results fed into clinical audit cycles Grants obtained with local researchers as lead DPDM expanded to second institution Further publications from DPDM graduates	Kenyan-run NGO with links to LSTM through Board of Trustees and collaborative research projects Over 500 HIV counselling and testing sites established Programme twinned with other countries in SSA. Research findings incorporated in international policy	REACH Trust - Independent Malawian research Trust established with Board of Trustees and Malawian Director Diverse funding and research portfolio. Ongoing advocacy with MoH and policy contributions.	Fully DRC run with global links to funders and academics
*Timing (months from start)*	*(48-84)*	*(6-120)*	*(60-120)*	*(12-192)*

*Abbreviations*

DPDM Diploma in Project Design and Management

EQUINET The Network on Equity in Health in Southern Africa

IPASC Institut Panafricain de Santé Communautaire

KATH Komfo Anokye Teaching Hospital

KNUST Kwame Nkrumah University of Science and Technology

LSTM Liverpool School of Tropical Medicine

LVCT Liverpool VCT, Treatment and Care

MoH Ministry of Health

NGO Non-governmental organisation

NTP Malawi National Tuberculosis Control Programme

REACH Research for Equity And Community Health Trust

#### Role of funding source

None

#### Ethics committee approval

Not required.

## Results

### Commonalities between case studies (table 2)

**Table 2:** Generic monitoring indicators for capacity building programmes derived from commonalities in case studies

Phase	Common activities	Generic indicators derived from activities	Examples of sources of evidence for indicators used in case studies
Awareness	Lack of local capacity recognised early in project Stakeholders agree to support activities to address capacity gaps Need for uptake of research outputs identified	List of capacity gaps to be filled List of stakeholders who will be critical for implementing project outputs Evidence of engagement of stakeholders (beyond core project team) able to facilitate capacity building activities	Written assessment of gaps in capacity Notes of meetings with stakeholders beyond research team (e.g. government or institutional directors)

Experiential	Capacity building activities focused primarily on individuals directly involved in project Formal and informal routes for using project outputs to influence policy/guidelines are explored Formal plans for addressing capacity gaps are gradually defined Preliminary models for capacity building are tested and adapted for scale up Strategies for ensuring that the relevant policies were in place or updated	Written plan and timescale for addressing gaps agreed with stakeholders Documented strategy for using project outputs to rectify mismatches/gaps between evidence and policy/practice Results of testing of pilot projects/models for capacity building	Annual plans with targets, timescale and details for rectifying policy gaps Review of comparison of different models and report of testing of models

Expansion	Concerted effort to influence policies and practice Focus broadens from individuals to strengthening institutions and systems Capacity building activities and individuals expand and begin to be integrated in existing structures Researchers inputs down-scaled to provide light touch guidance Sustainable funding actively sought Peer-reviewed publications from research and capacity building published	Expanded relevant skills and workforce Reduction of inputs by northern partners Regular review process instigated for updating/developing relevant policies Evidence of strengthening of systems (e.g. new committees or reporting structures) Diversification of funding sources independent of original funders Publications and/or presentations at national/international meetings	Training records indicating number of individuals trained, topics covered, skills audit and evidence of use of new skills Individual student assessments to demonstrate knowledge, skills and competencies Institutional annual budgets showing earmarked research funds Workplan showing phase out of northern partners, policy review and set up of new structures Documentation of number, type and success rates of publications and funding applications

Consolidation	Expansion beyond initial project objectives and original institution/region/country Southern partners lead bids for alternative sources of funding independent of original project funds Southern partners responsible for project and budget management	Evidence that long-term funding has been secured Project management and key decisions, such as commissioning of further external inputs, led by southern partners	Financial statements showing diverse sources of funds and that southern institution is responsible for budgeting Minutes of meetings showing key decision-making by southern partners

In all the projects a need for capacity building had been identified early in the '*awareness' *phase and efforts had been made to engage relevant non-academic stakeholders including policy makers and service providers. For example, in CS2 it was recognised that the number of facilities needed to be increased and more staff needed training in HIV testing and counselling before HIV services could be scaled up. In CS3 a partnership was formed between the national TB programme and universities in Malawi and the UK to make sure that the research would address the national priorities for TB services.

During the *experiential *phase, plans for capacity building were developed in collaboration with stakeholders, and implementation was started. Mechanisms for demonstrating international credibility such as quality systems and audits were instigated. In Ghana (CS1) the teaching hospital substantially increased internet availability, set up a research office and provided seed funds for projects. In DRC an institute was established to provide training in community health (CS4).

In the *expansion *phase the new capacity generated by successful activities was embedded in existing structures and there was evidence of innovation. There was also a concerted effort to influence policies and to identify funding that would support core services and therefore promote sustainability. For example, HIV services were extended to include post rape care and services for vulnerable groups, and national guidelines were produced (CS2). The training institute in DRC became part of an international social science network (CS4).

By the final *consolidation *phase the inputs by external partners were minimal, capacity building activities had been incorporated into routine processes, and independent funding, including for core functions, secured. For the purposes of our analysis, entry into this consolidation phase was considered to be evidence of sustainability. For example, by this phase Ghanaian tutors were completely responsible for running a research skills course, and local researchers had obtained their own grants (CS1). Projects in Kenya and Malawi had established themselves as independent non-governmental organisations (CS2 and CS3), and two projects been awarded international collaborative grants (CS2 and CS4).

Underpinning all the projects was a strong emphasis on mentorship and on creating opportunities for networking [14,15]. Interestingly all the projects had set up rigorous systems for monitoring and evaluating quality to demonstrate the credibility of their capacity building activities, and had published their capacity building achievements suggesting that the project team had transferred their research expertise into the field of capacity building. Due to lack of detail in project budgets, it was not possible to extract information from the case studies about the funds devoted to monitoring and evaluation. All the projects had promoted ownership by southern partners from the start and had explicit strategies for reducing reliance on northern partners. In addition, the projects all faced similar challenges in achieving sustainability. These were:

• high turnover of staff and stakeholders which necessitated regular re-engagement and briefing of individuals often in many different locations

• integrating new initiatives into existing systems

• ensuring that new skills and staff were utilised effectively

• identifying and securing sources of sustainable funding

• using evidence from the projects to influence policy

### Differences between case studies

The case studies involved different tiers of the health system varying from a national disease control programme (CS3) and a tertiary hospital (CS1) to community clinics (CS2) and included examples of governmental and non-governmental organisations. Although all the case studies originated from a research project, only one or two members of the original research team were involved in the capacity building components. As projects developed they gradually drew in a wide variety of additional stakeholders including policy makers (CS2 and 3), administrators (CS1), information technology specialists (CS1), laboratory staff (CS2 and 3), health providers, community members and various professional organisations (CS1-4). Although all the case studies incorporated an external review, the 'reviewers' ranged from external examiners (CS1) to members of advisory or management groups (CS2, 3 and 4).

The sources of sustainable funding that were eventually secured included money from central government's training budgets (CS1), contributions from project participants towards the cost of courses (CS1, CS2, CS4), income from selling consultancy services (CS2), and externally funded research grants (CS3, CS4). In most projects, southern partners had invited northern partners to continue to have limited but well-defined inputs to strengthening capacity such as tutor training (CS1, CS2), or as collaborators on research proposals (CS2, CS3). In some instances the capacity building was led by individuals who were not part of the original project team (CS1). All projects had expanded to incorporate additional institutions (CS1) or countries (CS2, CS3, CS4).

The time period covered by each of these case studies ranged from 84 to 192 months (median 120 months) and because there was no clear transition point between phases the following times are rough estimates. The time taken to become sustainable (i.e. to reach the consolidation phase) was 60-192 months (median 66 months) (table 3). The median time (range) taken for projects to progress through the awareness stage was 15 (1-36) months, with 25.5 (12-84) months for the experiential phase and 30 (17-44) months for the expansion phase. The duration of these phases was highly variable and was influenced by many factors including the amount of funding, rate of staff turnover, political instability (CS4) and the amount of harmonisation necessary to embed activities in existing systems.

**Table 3:** Number of months spent on each phase of projects

Project phase	CS1	CS2	CS3	CS4	Median
Awareness	12	18	1	36	15

Experiential	27	24	12	84	25.5

Expansion	36	42	20	84	39

Consolidation	36	114	60	180	87

Total follow up time	84	120	120	192	120

**Time to reach consolidation phase (i.e. to become sustainable)**	**60**	**72**	**60**	**192**	**66**

### Monitoring indicators

Generic indicators were derived from project indicators that were used to monitor activities that were common to all case studies and relevant for sustainable capacity building. This commonality meant that they would be transferable between different projects and could be used to monitor progress towards building sustainable capacity. These indicators focused on increasingly complex measurements of capacity as projects matured. For example early project indicators often included evidence of engagement of stakeholders, such as minutes of meetings showing that stakeholders had participated in the meetings. Indicators in more mature projects provided evidence that stakeholders were making critical decisions such as commissioning external inputs. Examples of the types of evidence used in the case studies to demonstrate that these indicators had been achieved are provided in table 2. Each project also had its own unique indicators that were not transferable to different contexts. Examples of early stage unique indicators included turnaround times for marking assignments, number of sites providing services and use of feedback to improve a curriculum. Later stage unique indicators included changes in particular behaviours (e.g. willingness of trainees to contribute to course fees; professional attitude to HIV clients) and strengthening of institutional functions such as ethics committees, and governance and financial accountability systems.

## Discussion

Analysis of commonalities and differences between these case studies has enabled us to identify indicators and associated evidence that suggest a good likelihood that new capacity will be sustainable. The generic indicators were transferable across projects. Examples of these generic indicators and how they became more complex as projects matured is illustrated by the following list of evidence generated over time by each project. Evidence:

• of early engagement of key stakeholders

• of a skills audit

• that the research addressed policy gaps

• that robust funding for core services had been secured

• that project management and key decisions were led by southern partners.

Indicators that are generic to all projects can be combined with those that are unique (i.e. are not transferable) to individual projects thereby creating the possibility of developing a tool for monitoring progress in capacity building that could be applied prospectively and adapted for projects in different contexts. Because the tool includes both generic and unique project-specific indicators it could be tailored for projects in different settings and at different stages of maturity. To facilitate comparability between projects the tool needs to be revised as projects mature and only projects at approximately similar stages of maturity should be compared with each other.

Indicators from the final consolidation phase could be used for an end-of-project evaluation. For example in CS1 evidence of the sustainability of the capacity that had been built could include financial statements demonstrating secure funding for core services and research, evidence from external reviews that course adaptations improved quality and met international standards, course revisions showing new innovations, timetables indicating that all teaching is done by local tutors, and course graduates leading new grants and publications.

The indicators derived from our case studies became more complex and sophisticated as the projects developed. This corroborates previous suggestions that monitoring of the early stages of capacity building should be much more 'light touch' than the later stages when more sophisticated capacity such empowerment and changes in systems should be monitored. Although capabilities such as resilience, innovation, motivation and credibility are needed to achieve this level of capacity development [5] our indicators did not specifically monitor these capabilities. Thoughtful timing and design of monitoring and evaluation mechanisms is important to avoid introducing overly complex systems too early in a programme as these could lead to collapse of the monitoring process [5]. Our finding, that it takes over 5 years for projects to start to become sustainable, corroborates published information [5]. Funders need to be aware that this long time scale combined with the pro-active management that needed to regularly refine monitoring indicators and collect evidence, means that significant human and financial resources are required to demonstrate that sustainable capacity building has been achieved. More detail in project reports about the costs of monitoring and evaluation would assist in planning explicit resource allocation for these activities.

In our case studies, capacity building was considered sustainable when the developing country institutions were able to manage the project, to source funds for core activities and to adapt and innovate by themselves without relying on northern partners. To effectively build capacity it is important to be able to create partnerships with a range of decision makers [16] and in all of our case studies there was evidence of ongoing evolution of new stakeholder partnerships. All the projects continued to adapt and expand long after the original objectives had been achieved, highlighting the adaptability, resilience and motivation of the southern partners. Thus although the point at which the projects were no longer reliant on northern inputs was reasonably well defined, constant evolution, adaptation and expansion of projects meant that there was no clear end-point to the capacity building activities. We identified indicators of sustainability retrospectively by analysing case studies which had demonstrated that they were sustainable. It will therefore be important to prospectively test whether these indicators are useful predictors of the ability of programmes to achieve sustainable capacity in the long-term and whether the indicators are helpful for identifying reasons why programmes may not be sustainable.

It is possible that by taking case studies from our own experience we may have biased the results. However cases were selected using pre-determined criteria, which were based on evidence from the literature and seven cases were rejected because they did not fully meet these criteria. Our close involvement with the selected cases enabled us to bring a depth of knowledge and understanding to the analysis that would not be possible for an independent reviewer. Although we only included four case studies, the fact that there were so many commonalities between them suggests that our process identified the major indicators that were appropriate for a range of contexts. These sustainability indicators for capacity building need to be tested prospectively in a variety of projects in order to evaluate their usefulness. This external and independent testing would also demonstrate whether we may have missed any relevant indicators by using a pre-existing framework or by being closely involved in the case studies.

Despite significant investment in capacity building in developing countries, and an extensive literature concerning theoretical evaluation tools, published examples of real-life evaluations of the sustainability of capacity building are almost non-existent. We have shown that indicators for these evaluations need to be developed in collaboration with stakeholders to promote 'buy-in', and they should be revised regularly so that they can evolve with the project. Monitoring tools which are inflexible and based on assumptions, could stifle innovation, alienate the project team and eventually constrain, rather than enhance, capacity building activities. For example, a common reporting requirement is the number of workshop participants, an indicator which promotes high volume potentially at the expense of quality, whereas a more appropriate indicator may relate to the acquisition and use of new skills by a smaller number of individuals. Our case study analysis has identified transferable generic indicators which can be combined with unique project-specific indicators and used flexibly for monitoring and evaluating capacity building.

## Conclusions

Key lessons from our research about monitoring and evaluating capacity building are

1. Generic (common to all projects) and context-specific (unique to each project) indicators can be combined and tailored to provide a tool for monitoring and evaluating the success and potential sustainability of capacity building efforts

2. These indicators need to increase in sophistication as projects mature. The use of overly complex systems too early in a project may lead to resistance and collapse of the monitoring process

3. Indicators for monitoring capacity building need to have 'buy-in' from stakeholders and should be revised regularly as assumption-based, inflexible monitoring frameworks stifle innovation and risk alienating the project team

4. It takes 5-10 years for projects to become sustainable and significant human and financial resources are required to carry out the rigorous, in-depth evaluations needed to demonstrate the effectiveness of investments in capacity building

## Competing interests

The authors declare that they have no competing interests.

## Authors' contributions

All authors contributed to and approved the final version of the paper. IB conceived the study design, contributed to data extraction, produced the first draft of the paper and finalised manuscript for submission. MT and ST contributed to study design, data extraction for all case studies and all manuscript drafts. SBS, DA, BNS and AB contributed to study design, data extraction for their own case studies and several manuscript drafts.
